# Representing annotation compositionality and provenance for the Semantic Web

**DOI:** 10.1186/2041-1480-4-38

**Published:** 2013-11-22

**Authors:** Kevin M Livingston, Michael Bada, Lawrence E Hunter, Karin Verspoor

**Affiliations:** 1 Department of Pharmacology, University of Colorado Anschutz Medical Campus, Aurora, CO, USA; 2 National ICT Australia, Victoria Research Laboratory, Melbourne, VIC, 3010, Australia; 3 Department of Computing and Information Systems, The University of Melbourne, Melbourne 3010 VIC, Australia

**Keywords:** Ontology, Conceptual data modeling, Annotation, Markup, Provenance, OWL, RDF

## Abstract

**Background:**

Though the annotation of digital artifacts with metadata has a long history, the bulk of that work focuses on the association of single terms or concepts to single targets. As annotation efforts expand to capture more complex information, annotations will need to be able to refer to knowledge structures formally defined in terms of more atomic knowledge structures. Existing provenance efforts in the Semantic Web domain primarily focus on tracking provenance at the level of whole triples and do not provide enough detail to track how individual triple elements of annotations were derived from triple elements of other annotations.

**Results:**

We present a task- and domain-independent ontological model for capturing annotations and their linkage to their denoted knowledge representations, which can be singular concepts or more complex sets of assertions. We have implemented this model as an extension of the Information Artifact Ontology in OWL and made it freely available, and we show how it can be integrated with several prominent annotation and provenance models. We present several application areas for the model, ranging from linguistic annotation of text to the annotation of disease-associations in genome sequences.

**Conclusions:**

With this model, progressively more complex annotations can be composed from other annotations, and the provenance of compositional annotations can be represented at the annotation level or at the level of individual elements of the RDF triples composing the annotations. This in turn allows for progressively richer annotations to be constructed from previous annotation efforts, the precise provenance recording of which facilitates evidence-based inference and error tracking.

## Background

Annotation of artifacts such as documents and images with metadata is a scholarly practice with a long history. A wide variety of annotations have been represented in a wide range of formats. In the bulk of that work, each annotation consists of a basic association of one conceptual resource (*e.g.*, an ontology class, schema element, database identifier) with one target (*e.g.*, document, text span, database entry) via an explicit or implicit relationship. Single-concept annotations have proven very useful, for example, in computing term enrichment [1] or for indexing for search [2]; however, they do not provide a detailed representation of the content they are describing. As information needs increase and annotation efforts expand to capture more complex information, complex knowledge structures formally defined in terms of more atomic knowledge structures will need to be represented.

Where they exist, more structured annotations tend to be represented in *ad hoc* formats suited to one particular type of annotation or task but are not broadly applicable or interoperable. Several prominent annotation models not limited to specific types of tasks or information have been created, and components that enable annotations to denote knowledge structures more complex than atomic concepts have been added very recently to these models [3,4]. Yet there have been no mechanisms put forth by which these more complex annotations can refer to other annotations and by which their provenance can be unambiguously recorded. There have also been prominent efforts in scientific workflow provenance [5,6]. That work, however, primarily focuses on annotating experimental data, typically annotating lists of identifiers or numeric data with their origins, not on annotating with dynamically composed and compositional knowledge structures.

An effective annotation model, in addition to being applicable to many annotation use cases and supporting the specification of complex knowledge structures, needs to be able to unambiguously represent annotation provenance. While ontologies strive to be complete, it is likely that specific applications will require dynamic construction of concepts, either through data-driven methods [7] or compositional concept formation [8]. To support and document the provenance of these more complex annotations, annotators (both human and computational) need the ability to refer to existing annotations as the basis of more complex annotations. For example, in the linguistic domain, an annotation representing part of a syntactic parse tree may wish to build upon existing token or part-of-speech annotations. Similarly, in the biomedical domain, a protein interaction event annotation may wish to leverage existing annotations identifying specific proteins. As annotation efforts become more ambitious, they will naturally build upon previous annotation efforts, and tracking the provenance of constructed knowledge representations being used for annotations at a fine-grained level will be important to facilitate inference and error analysis.

This paper proposes a task- and domain-independent formal ontological model for the creation of annotations and their linkage to their denoted knowledge representations, which can be singular concepts or more complex knowledge in the form of sets of RDF assertions. With this model, progressively more complex annotations can be composed from other annotations, and this provenance can be unambiguously represented at either a coarse- or fine-grained level. We have designed our annotation model to be generic so as to facilitate the concurrent use of multiple types of annotations (*e.g.*, syntactic annotation and semantic annotation). Additionally, it allows for the creation of arbitrarily complex annotations, both in terms of their denoted knowledge and of any other annotations upon which they rely. All of this information can be losslessly recorded, facilitating inference and error tracking in large computational annotation efforts. We have implemented this model as an extension of the Information Artifact Ontology in OWL and made it freely available. We also show how it can be integrated with several other prominent generic annotation models.

## Results

### Overview

A central aspect of our model is the capability to accurately capture the provenance of annotations, in terms of precursor annotations, created by a human or computational annotator. The annotation model we present here is generally applicable to arbitrarily complex, structured annotations applied to any content in any context. It is not specific to text annotations, although our primary use cases are related to understanding biomedical text.

Our model provides two key contributions above existing annotation and provenance models. First, we provide a generic model for complex and compositional annotations that extends existing general-purpose annotation models. Second, we provide a model for documenting the provenance of the construction of the triples used as the denoted knowledge representations by these annotations. Our model goes beyond modeling the provenance of whole triples (for which there are sufficient existing methods, as discussed in the Related Work section) and extends the provenance modeling to document the source of individual statement elements that are used to construct triples.

This proposal is neutral with respect to annotation template; *i.e.*, the choice of terminologies, ontologies, or schemas used for annotation and the nature of the denoted knowledge representations is left to the annotator. Several existing annotation models handle the association of annotations to text or other targets, and we discuss the integration of their representations with our model in Additional file 1 and Additional file 2. Additionally, as this proposal focuses on the linkage of annotations to their denoted knowledge representations and on the provenance of these knowledge representations, details about the recording of other types of annotation metadata such as author and creation date (for which there are existing proposals, *e.g.*, [3,4,9]) are largely elided from this paper. Finally, our ontological model is neutral with respect to the methodology by which any such annotations are created.

We reuse or extend existing community-curated ontologies where possible, and we therefore present our proposal as an extension of the Information Artifact Ontology (IAO), which is a member of the Open Biomedical Ontologies library of ontologies [10] (though not all of the concepts of these ontologies are specific to the biomedical realm). The IAO focuses on the representation of types of *information content entities*, which are defined to stand “in relation of aboutness” to other entities; that is, an information content entity is in some way “about” some other concept(s). For example, within the biomedical domain, data, images, and text are all in some way about sets of biomedical concepts. The IAO provides a hierarchy of types of information content entities as well as types of aboutness, including *denotation*, in which the information content entity specifically refers to some other concept (*e.g.,* the word “apple” denotes either a specific apple or the more general concept of an apple). We hold that an annotation is a type of information content entity, as it is in some way about the entity it is annotating. We are engaged in the ongoing process of submitting our model to the IAO for inclusion. An OWL representation of our model as an extension of the IAO is provided in Additional file 3.

### Namespace and notation

Our in-house knowledge base of biomedicine (KaBOB) is the aggregator of our work. KaBOB extensions of an ontology are named by prefixing the ontology’s namespace with the letter ‘k’; the namespace kiao: is therefore used for our extension of the IAO (whose namespace is iao:), and the ex: namespace is used for examples. In this document, fixed-width font will be used to identify concepts. Class names begin with a capital letter, while instance and property names begin with a lowercase letter. Additionally, instances are named mnemonically with letters corresponding to their class names; *e.g.,* instances of the class RdfResourceAnnotation have names starting with “ra”. RDF triples and quads are presented using an abbreviated n-triple/quad format for readability, using name-space-abbreviation:local-name instead of full URIs.

### Representation of annotations

We have created a top-level Annotation class, defining an annotation as an information content entity that is used to concisely describe, comment on, or otherwise make an assertion or set of assertions about an existing information content entity. Thus, for example, a linguistic part-of-speech tag can be used to annotate a word within a piece of text to describe its syntactic or morphological behavior; a Java keyword (*e.g.*, @deprecated) can be used to annotate a segment of Java source code to specify a property of a Java class, method, variable, parameter, or package; and a GO term can be used to annotate a digital representation of a gene or gene product to make an assertion about some aspect of the biological functionality of the latter. Conciseness seems to be a common trait among the many types of annotations we have considered, so, *e.g.*, a book written about a poem would seem to be beyond the bounds of what most would consider an annotation. Additionally, an annotation provides additional information about an entity but is typically not fundamental to the entity; therefore, we would not consider a title of a journal article to be an annotation of the article: Even though it concisely describes an existing information content entity, *i.e.*, the body of the journal article, it is a canonically required part of the article. Furthermore, an annotation is typically either incorporated into the entity (*e.g.*, in the classical case of annotation of writing in the margins of a book, which becomes a physical part of the book) or can be otherwise retrieved along with the entity it is annotating (*e.g.*, in the case of GO-term annotations of database entries of proteins).

A subclass of Annotation could be defined for any type of information content entity used to annotate another entity (*e.g.*, PartOfSpeechTagAnnotation, JavaKeywordAnnotation, GoTermAnnotation). However, since we are motivated toward utility for the Semantic Web, we introduce only two subclasses, RdfResourceAnnotation and RdfGraphAnnotation, representing RDF resources and graphs, respectively, that are used to annotate other information content entities. These two subclasses should be all that is needed for the representation of annotations in RDF stores, in which everything should be an RDF resource or graph. Furthermore, as long as information content entities used for annotation are offered as RDF constructs (so that they can be used in RDF stores), other annotation subclasses should not be needed for their representation in RDF stores. For example, since GO terms are also offered as RDF resources, GO-term annotations can be stored as instances of RdfResourceAnnotation, obviating the need for a GoTermAnnotation class (unless there is further desired axiomatization for GO-term annotations).

### Resource annotations

In our model, a *resource annotation* is an annotation that associates a single rdfs:Resource with a target. A resource annotation is modeled as rdf:type kiao:RdfResourceAnnotation. The relation iao:denotes is used to associate a given annotation with the concept being used to annotate the target. This property relates an information content entity (in this case a resource annotation) to something to which it is specifically intended to refer.

One of the primary types of text annotation is syntactic annotation, which is often produced by text mining systems (*e.g.*, [11]). To demonstrate the applicability of our model to syntactic annotation, we use a fragment of the example sentence used by Liu *et al.* in their study of dependency parsing for information extraction [12], *i.e.*, the phrase “Interferons inhibit activation of STAT6”. (For the purposes of an example, we have taken some liberty in creating example classes and relations that we believe are faithful to the native dependency parse representations [13].)

**Figure 1:**
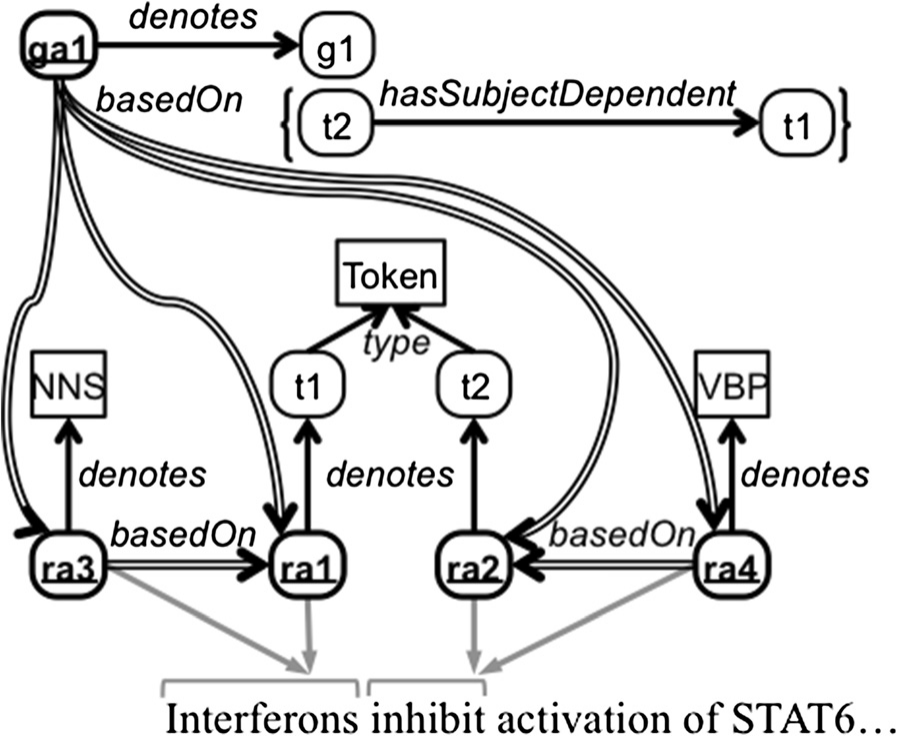
**Example syntactic annotations.** This figure depicts five syntactic annotations as bold ovals with underlined labels: four RdfResourceAnnotation instances, each with the prefix “ra”, and one RdfGraphAnnotation instance, prefixed with “ga”. Rectangles represent classes, while instances have rounded corners. Double-lined arrows depict basedOn assertions. Thin gray arrows are used to provide reference to the text, although their representation is elided in this paper. The statements inside brackets are contained within the corresponding RDF graph.

Common tasks at the beginning of text mining pipelines include tokenization and part-of-speech tagging [14]. Figure 1 depicts four resource annotations: ra1, ra2, ra3, and ra4. The concepts in the object positions of the denotes assertions are part of the domain model used by the annotator and are not part of the proposed annotation model itself. ra1 and ra2 denote specific instances of tokens (represented here as instances of the class Token), while ra3 and ra4 denote plural nouns and singular present-tense verbs, respectively (represented here by their Penn Treebank part-of-speech tags [15]). In this example, the annotator made the domain-specific representational choice to model the tokens as instances so that they can be specifically referred to later by subsequent annotations, as will be shown in the next section. Abstract relations connecting the resource annotations to text spans are shown in Figure 1 as gray arrows, with gray brackets representing the text spans. Existing models for linking annotations to the object being annotated can be used with our model, for example, the relations oa:hasTarget[3] or ao:context[4] could be used to model these gray arrows. As our model is neutral relative to these representational decisions, this aspect of modeling the example annotations is elided from this document for simplicity and clarity.

The following are RDF triples representing two of these annotations, asserting that ra1 and ra3 are resource annotations that denote a particular instance of a token (represented here as t1) and plural nouns (represented here as NNS), respectively.

**Figure 2:**
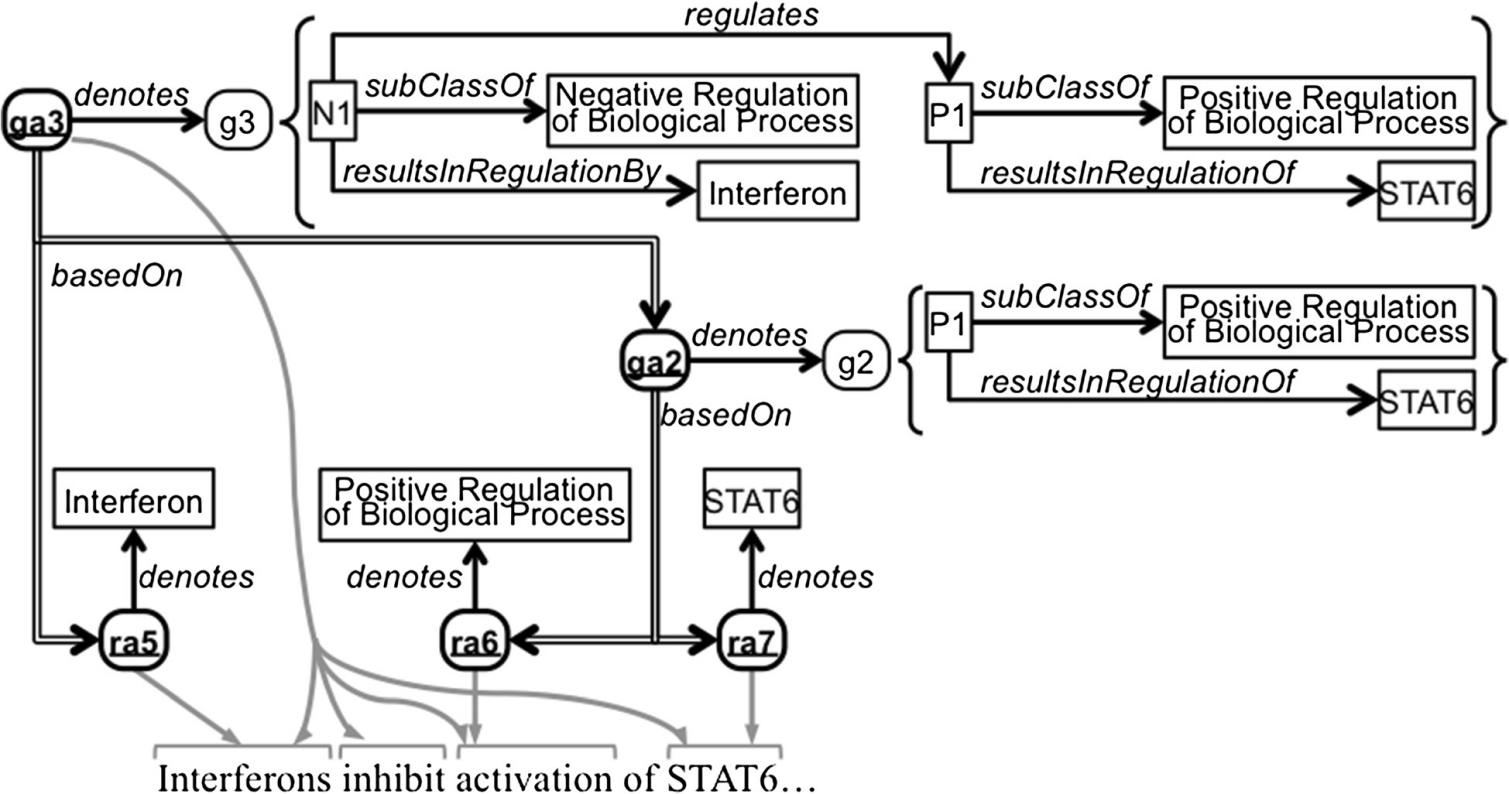
**Example biomedical semantic annotations.** This figure depicts five semantic annotations as bold ovals with underlined labels: three RdfResourceAnnotation instances and two RdfGraphAnnotation instances. (See the caption of Figure 1 for explanation of shapes and arrows).

Semantic annotations of text fragments, such as those in the CRAFT Corpus [16], are another primary use case for the model presented here. In Figure 2, the example sentence fragment from Figure 1 has been annotated with semantic classes in the manner of CRAFT annotation. (In some cases, we have used ontologies and classes not used in CRAFT in order to simplify the biology and therefore the example.) The biomedical classes and properties used to model the examples in this paper are not part of the proposed annotation model. In Figure 2, the three example resource annotations ra5, ra6, and ra7 denote relevant biological concepts: ra5 denotes interferons, a group of proteins represented here by Interferon (IPR000471) in the InterPro database of protein sequence signatures and families [17]; ra6 denotes the upregulation of biological processes, represented here by positive regulation of biological process (GO:0048518)^a^ in the Gene Ontology [18]; and ra7 denotes STAT6 proteins, represented here by STAT6 (PR:000001933) in the Protein Ontology [19]. The following are RDF triples for two of these annotations, specifically asserting that ra6 and ra7 are resource annotations that denote positive regulation of biological processes (represented here as GO:0048518) and STAT6 proteins (represented here as PR:000001933), respectively.

### Graph annotations

While a resource annotation relies on a single RDF resource for annotation, a *graph annotation* is an RDF graph, composed of a set of one or more RDF statements, that is being used to annotate another information content entity. A graph annotation is modeled as rdf:type kiao:RdfGraphAnnotation. A graph annotation is connected to a named graph of RDF statements using the property iao:denotes. While a graph annotation is directly linked to a named graph, it actually denotes the *content* of the named graph (*i.e.*, the RDF graph that the named graph encodes or represents) and not the named graph itself; this is consistent with the semantics of named graphs proposed by Carroll *et al.*[20], which states that any assertion in RDF about the graph structure of a named graph is understood to refer to the underlying RDF graph. As before, the nature of the denoted knowledge representations (*i.e.,* the set of RDF statements) is left to the user, as our metamodel focuses on the linkage of annotations to such representations and, as presented in the next section, the provenance of compositional annotations.

Linguistic annotation is frequently done in a pipeline where subsequent stages build upon the annotations produced by earlier stages. In addition to the aforementioned resource annotations, Figures 1 and 2 depict several RdfGraphAnnotation instances. In Figure 1, there is one graph annotation that denotes the subject dependency of token t2 on token t1, represented here as a graph (g1) containing one RDF triple with subject t2, property hasSubjectDependent, and object t1. The following are triples/quads for graph annotation ga1:

Since the annotator made the domain-specific modeling choice to represent resource annotations ra1 and ra2 as denoting instances of tokens, a dependency assertion among them (*i.e.*, that token t2 syntactically depends on token t1 as the subject of the sentence, as seen in Figure 1) was able to be created. If the annotator had pointed these resource annotations directly to the class Token (analogous to the direct pointing of resource annotations ra3 and ra4 to the classes NNS and VBP, respectively), then it could only have been asserted at the class level that Token hasSubjectDependent Token rather than the assertions relating the specific tokens. It is important to remember that this representation of syntactic dependency is of our own choosing for this example and that any user of our metamodel of annotations is free to represent syntactic dependency (or any other knowledge denoted by the annotations) as he chooses.

Semantic concept annotation, such as the manual annotation performed on the CRAFT Corpus or annotations created by text mining systems, can also be built in layers. Figure 2 depicts two RdfGraphAnnotation instances ga2 and ga3. The former denotes the positive regulation of STAT6 protein, represented here as a graph g2 containing a dynamically constructed subclass P1 of the GO class representing positive regulation (GO:0048518) in which STAT6 (PR:000001933) is regulated. The graph ga3 builds upon the denoted knowledge representation of ga2 and denotes the negative regulation of the positive regulation of STAT6 protein by an interferon, represented here as a dynamically constructed subclass N1 of the GO class representing negative regulation (GO:0048519) in which the regulating entity is an interferon (IPR000471) and the regulated process is a positive regulation of STAT6 protein. The following are triples/quads for graph annotation ga2:

The use of graphs has the advantage of separating the representation of the annotation from the representation of the denoted content and thus protects users of a given annotation from committing to or believing the propositions represented in the annotation unless desired. For example, a given annotation could denote the fact that STAT6 can bind calcium ions as one of its functionalities, which could be represented as, *e.g.*, one RDF triple (STAT6 hasFunction CalciumIonBinding) in an RDF graph. Since this triple is placed in its own graph, a reader of this annotation is not committed to believing that STAT6 can bind calcium ions; what has been effectively represented is that this particular annotation says that STAT6 can bind calcium ions. Just as our model is agnostic with respect to the denoted knowledge representations of annotations, we do not seek here to explicitly represent confidence, trust, or other epistemological or modal information; however, such information could be modeled independently and added orthogonally or compositionally to our proposed model.

### Provenance of compositional annotations

As annotations become more complex, tracking their provenance becomes increasingly important. Provenance tracking is necessary in order to know which other annotations were used in constructing an annotation and how their individual denoted representations were composed into larger knowledge structures. Additionally, provenance is needed for error analysis and blame attribution. For example, referring to the example in Figure 2, if ga2 is found to be incorrect only because it refers to the wrong protein, the error and blame should be properly attributed to the author of ra7, as the latter was the source of the incorrect protein identification. Likewise if ra7 is determined to be incorrect, annotations dependent on that annotation could be identified and retracted or updated as well.

Provenance information can be equally useful for disambiguation. Consider the case in which there are multiple competing annotations for the text “STAT6” (*e.g.,* those denoting human, mouse, and rat homologs of the STAT6 protein, which are represented as distinct entities in prominent biological repositories such as UniProt [21] but are all canonically referred to as “STAT6”). If, as in this example, there are multiple competing annotations for the specific type of protein but only one is used as the provenance for a larger annotation, such as ga2, then this provenance can be tracked to resolve the ambiguity caused by the competing annotations. This is one of the ways language understanding systems can successfully resolve ambiguity [22]. An annotation model such as ours that captures this information and provenance can be used to document these choices and facilitate error analysis.

In order to track provenance, we introduce the transitive relation kiao:basedOn, which is used to track both coarse-grained, annotation-level provenance and fine-grained, statement-element-level provenance in our model. We propose that this relation holds between two information content entities; therefore, the value of rdfs:domain and rdfs:range for this relation is iao:information content entity. Informally, kiao:basedOn holds between subject and object information content entities when the subject entity has been created relying in whole or in part on the already existing object entity. In this proposal, we are interested in making assertions of annotation provenance by recording that *specific annotations* have been created wholly or partly relying on other *specific annotations*. We make no restriction on the cardinality of kiao:basedOn, so a subject information content entity can be based on multiple object entities, and multiple subject information content entities can be based on the same object entity.

### Annotation-level provenance

The simplest way to record provenance is to make coarse-grained basedOn assertions between annotations. A basedOn statement can be made between two annotations either when there is a direct relationship between the annotations, such as one directly using one or more elements of another, or when there is an indirect relationship, such as one being used as the justification for another’s existence even though no part is explicitly shared (*e.g.,* an annotation of a text span with a specific protein class being used to justify an annotation of the same text span with the top-level class protein (PR:000000001) from the Protein Ontology).

Most syntactic dependency parsers use tokenization and part-of-speech tags produced by other annotation systems as input. Figure 1 depicts six different annotation-level basedOn assertions between syntactic resource annotations. Those from ra3 to ra1 and from ra4 to ra2 have been asserted because ra3 and ra4, denoting parts of speech, were created based on ra1 and ra2, denoting tokenization, respectively. The following triples represent the assertions of provenance among these four resource annotations:

Note that these assertions are made even though there are no direct relationships among the denoted tokens and parts of speech, *e.g.*, between the concepts denoted by ra3 and ra1 (*i.e.*, between plural nouns, represented here by the part-of-speech tag NNS, and token t1); however, ra1 was instrumental in the creation of ra3, and the basedOn statement documents this. The part of the syntactic dependency parse annotated by the graph annotation ga1 used both the tokenization annotations (ra1 and ra2) and the part-of-speech annotations (ra3 and ra4) to determine that token t1 is the subject of token t2, and thus ga1 is modeled with a basedOn relation to each of these four resource annotations. The following four triples represent the annotation-level provenance of graph annotation ga1:

Just as layers of annotation can build upon each other in syntactic annotation, semantic annotation can also be composed compositionally. Provenance relations are analogously depicted among the semantic annotations in Figure 2. Graph annotation ga2 was built using information from resource annotations ra6 and ra7. Similarly, the larger graph annotation ga3 records that it was built using information from resource annotation ra5 and from graph annotation ga2. The provenance information can be traced from annotation to annotation, and in this case one can see that ga3 is (partly) based on ga2, which in turn is based on ra6 and ra7. It is important to note that basedOn is general in that it can be used to create an annotation-level assertion of provenance from either a resource or graph annotation to a set of any combination of resource and/or graph annotations. Instances of RdfGraphAnnotation need not be strictly compositional; that is the statements in a graph annotation do not need to be based on other annotations and can incorporate new information not yet annotated elsewhere. For example, ga3 uses additional information not explicitly previously annotated, which is shown in Figure 2 by an additional gray arrow pointing to a segment of the text not previously annotated.

### Statement-element-level provenance

The second type of provenance represented in our model records detail at a more fine-grained level. Referring back to Figure 2, while the assertion ga2 basedOn ra7 is sufficient to model that at least some part of ga2 was based on ra7, it does not capture *which* elements of ga2 are based on ra7. Analogously, in Figure 1, the graph annotation ga1 documents that it is based on resource annotation ra1 but nothing more specific than this. If the author of ga1 wishes to document that the object element (which denotes token t1) of the RDF statement of ga1 is based on ra1 (which also denotes token t1), then recording provenance at the annotation level is insufficient. In addition to documenting how compositional annotations were constructed for understanding or training purposes, this type of provenance is necessary to perform detailed error analysis.

**Figure 3:**
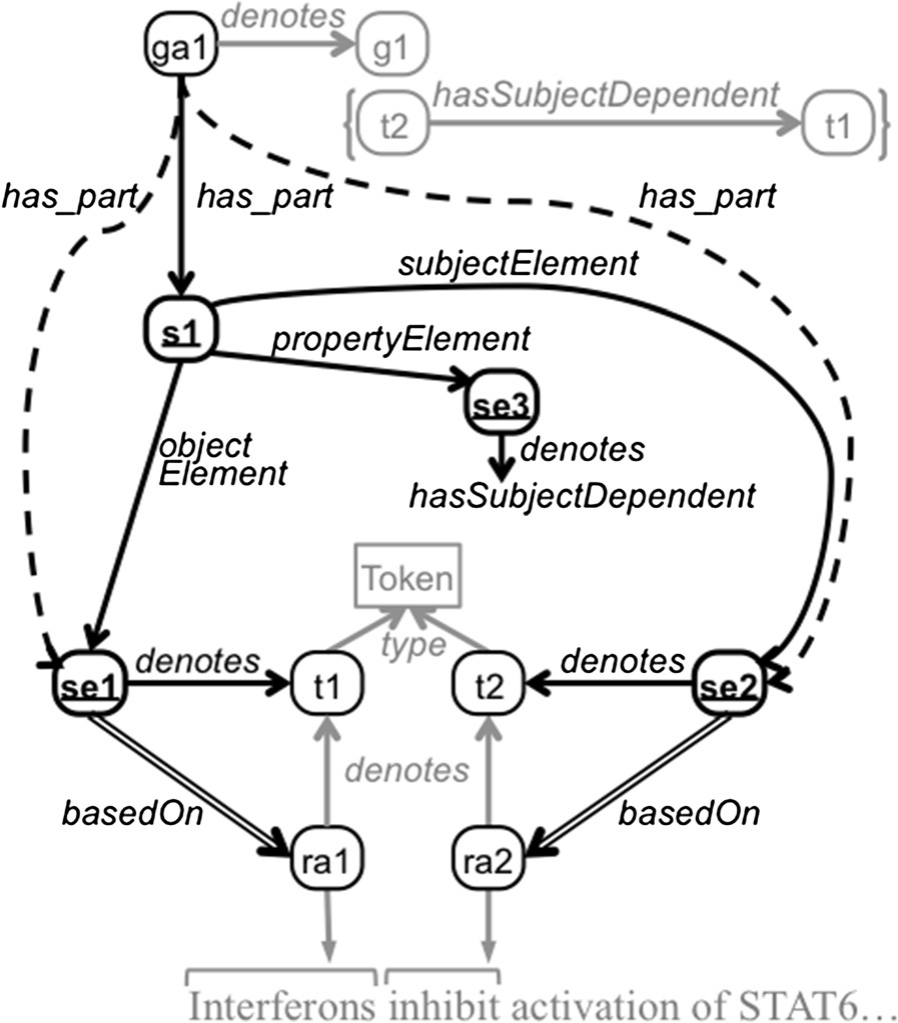
**Example of statement-element provenance.** This figure depicts an example of statement-element-level provenance. The RdfStatement and the three RdfStatementElement instances have bold ovals and underlined labels. This figure is an extension of Figure 1, and some of the parts of that figure have been preserved here but grayed out. Dashed lines show assertions that can be inferred. (See the caption of Figure 1 for explanation of shapes and arrows).

In RDF, the typical way to make statements about statements is to reify the statement itself as an instance of rdf:Statement. An RDF statement identifies its subject, property, and object via the relations rdf:subject, rdf:property, and rdf:object, respectively. However, RDF statements and their elements are conceptual representations; for example, in Figure 1, the RDF statement t2 hasSubjectDependent t1 represents the assertion that token t2 has as its subject token t1. To explicitly represent RDF statements as information content entities, we introduce the class kiao:RdfStatement, which is rdfs:subClassOf iao:information content entity. An example of a reified kiao:RdfStatement is the instance s1 in Figure 3. A graph annotation can then be connected to each reified statement of the graph annotation using the property obo:has_part.

In order to record the provenance about individual parts of a statement, these parts must also be reified as instances of kiao:RdfStatementElement. A reified statement is linked to its component instances of kiao:RdfStatementElement using three properties that mirror the properties used to reify the rdf:Statement itself (*i.e.,*rdf:subject, rdf:property, and rdf:object): kiao:subjectElement, kiao:propertyElement, and kiao:objectElement, each of which is rdfs:subPropertyOf obo:has_part; that is, a reified statement has these subject, property, and object elements as parts.^c^ Two reified instances of kiao:RdfStatementElement, se1 and se2, can be seen in Figure 3. The corresponding iao:denotes assertions from these statement elements to their denoted concepts (*i.e.,* tokens t1 and t2, respectively) are also depicted.

With an assertion from a graph annotation to a statement via obo:has_part and another assertion from the statement to a statement element via kiao:subjectElement, kiao:propertyElement, or kiao:objectElement (each a subproperty of obo:has_part), an obo:has_part assertion from the graph annotation to the statement element can also be inferred. The following axiom holds for subject elements, and corresponding axioms hold for property and object elements:

This is simply applying obo:has_part transitively. Figure 3 shows two derived obo:has_part assertions: Since graph annotation ga1 has_part statement s1, and s1 is linked to its component statement elements se1 and se2 via subjectElement and objectElement, respectively, it can be inferred that ga1 has these statement elements as parts.

Now that the reified statement has been decomposed into and appropriately linked to reified RdfStatementElement instances, the provenance of these individual pieces can be recorded. In the example in Figure 3, se1 is documented as being based on resource annotation ra1, and se2 is documented as being based on ra2. This is analogous to the use of the basedOn among annotations, except in this case the relation is being used among more fine-grained components of the model.

Our original model [23] used a more complex set of properties to record the same amount and type of information. The model proposed in this paper simplifies the representation of this information and the cognitive load of using the model significantly. The following triples represent statement s1 decomposed into statement elements se1, se2, and se3, along with the provenance of se1, as rendered in Figure 3:

The first two triples above represent that graph annotation ga1 has statement s1 as a part and that s1 is an RDF statement, and the next six triples decompose s1 into RdfStatementElement instances and specify their denotations. The relations subjectElement, propertyElement, and objectElement have an rdfs:domain of kiao:RdfStatement and an rdfs:range of kiao:RdfStatementElement, and thus type information can be inferred using RDFS reasoning, which we have omitted for conciseness. The seventh and eighth triples reify the object position of this statement, and the final triple documents that this statement element is based on resource annotation ra1. In this way, the annotator constructing ga1 can explicitly document the origin of every component piece.

**Figure 4:**
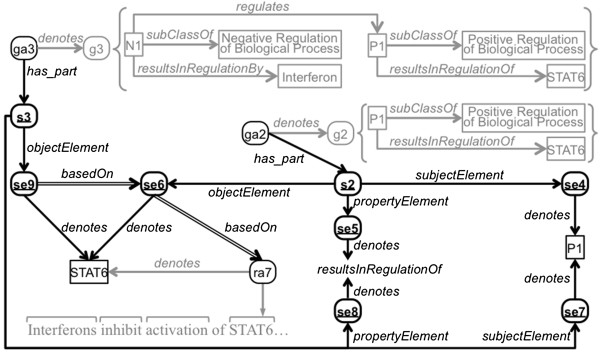
**Extended example of statement-element provenance.** This figure depicts an extended example of statement-element-level provenance. One RdfStatement from each graph and the six RdfStatementElement instances have bold ovals with underlined labels. This figure is an extension of Figure 2, and some of the parts of that figure have been preserved here but grayed out. Dashed lines show assertions that can be inferred. (See the caption of Figure 1 for explanation of shapes and arrows).

Just as RdfStatementElement instances can be based on instances of RdfResourceAnnotation, they can also be based on other instances of RdfStatementElement. As the composition of annotations becomes more complex and the layers of annotation get deeper, graph annotations will build on other graph annotations. This is especially true for annotations produced and used by computational language understanding systems [24]. For example, in Figure 4, statement element se9 is based on statement element se6, which is in turn based on resource annotation ra7. Figure 4 only shows the provenance of statement element se6 of statement s2 along with the provenance of statement element se9 of statement s3. Although not depicted, statement-element-level provenance could analogously be recorded for all elements of these statements, as well as for all statements of graph annotations ga2 and ga3. The following are triples representing annotation information for statements s2 and s3 and statement elements se6 and se9, including their provenance, rendered in Figure 4:

The first group of eight triples and the ninth triple above are exactly analogous to the triples used in the previous example. As in the previous example, here there is one reified statement (s2) that is part of a graph annotation (ga2). Analogously, the reified statement s3 is a part of graph annotation ga3, as can be seen in the third group of (eight) triples. However, in this example, there is an extended statement-element-level assertion of provenance: In the last triple, a statement element (se9) is asserted to be based on another statement element (se6), which was already created to partly document the provenance of a graph annotation (ga2). In this way, the annotator creating graph annotation ga3 can unambiguously document the specific element of the specific statement of graph annotation ga2 from which its reference to the protein STAT6 derives. This low-level provenance is essential for understanding the dependencies between layers of complex compositional annotations and for being able to unwind these layers to perform tasks such as error analysis and blame attribution.

## Discussion

### Use cases

In this section, we discuss types of tasks that our annotation model enables, along with specific examples of such tasks, including projects on which we are working as well as external efforts.

#### Integrating different types of annotations

A wide variety of annotation models and formats have been created for a wide range of tasks; however, the large majority of these are suited to one particular type of annotation or task and are not broadly applicable or interoperable. Our proposal is a generic metamodel of annotations and their linkage to their denoted knowledge representations. As such, it is neutral with respect to annotation template (*i.e.*, the choice of terminologies, ontologies, or schemas used for annotation), the nature of the denoted knowledge representations created, and the methodology by which annotations and denoted knowledge representations are created. As a result, our model can be generically used to integrate different types of annotations in a common representation, in turn leading to enhanced interoperability and queryability among these different types of annotations. Such integration is of substantial interest to us in the context of our efforts with the Colorado Richly Annotated Full-Text (CRAFT) Corpus, a collection of full-text biomedical journal articles that we have extensively marked up with a wide range of types of annotations, including those specifying mentions of biomedical concepts, coreference, discourse, as well as a variety of syntax, including sentence segmentation, tokenization, part-of-speech tagging, and Penn TreeBank tagging [16,25]. Relying on our generic metamodel, we are able to represent these disparate types of annotations and their denoted knowledge representations in a unified way. This, in turn, is required to enable matching of different types of annotations to various elements of formal natural-language patterns for automated understanding of biomedical text. This enables querying over multiple annotation types simultaneously, for example looking for all the noun-phrases that overlap with annotations to specific ontology terms, which might be a query for learning new vocabulary or patterns for identifying ontology terms in text.

Text is not the only artifact for aggregating multiple types of annotation. Tools such as the UCSC Genome Browser which layer annotation tracks over a visualization of the genome [26] demonstrate a clear need for integrating various types of genomic annotation, such as dbSNP [27], COSMIC [28], OMIM [29], and the GWAS catalog [30]. Use of our model would enable such annotations to be more easily integrated into such tool via a standard representation, and would further enable the integration of annotations to be usable beyond the scope of specific tools (*e.g.*, visualization) for new queries yet to envisioned by researchers.

#### Connecting annotations to the Semantic Web

Many types of annotations are represented in *ad hoc*, idiosyncratic formats (*e.g.*, Penn Treebank [15] for parts of speech, GAF 2.0 [31] for gene function, VCF [32] for SNPs) that, in addition to hindering interoperability, are obstacles to integration with the Semantic Web. We have implemented our annotation metamodel as a formal OWL ontology, and specific annotations are created as instances of relevant OWL classes. Consequently, linkage of these annotations and the data and knowledge they specify to existing ontologies [10], other RDF repositories [33], and the broader Semantic Web is considerably facilitated. Using our model, reasoning over annotation structure and their denoted semantics simultaneously is also enabled through the use of RDF- and OWL-based querying systems. For example, it is possible to query for annotations that are based on an annotation from a specific source that mention a subclass of a specific ontology term. Such a query might be used if a specific annotator is known to be highly accurate or inaccurate at a certain task. Such queries cannot be easily performed, if they can be performed at all, using combinations of tools on more idiosyncratic formats.

#### Linking annotations to arbitrarily complex knowledge representations

In most annotation efforts, each annotation consists of a basic association of one conceptual resource (*e.g.*, an ontology class, schema element, database identifier) with one target (*e.g.*, document, text span, database entry). However, as information needs increase and annotation efforts expand to capture more complex information, more complex knowledge structures will need to be represented. For example, although most GO annotations of genes/gene products straightforwardly link GO terms to database entries representing these genes/gene products, there has been a call to associate the biological-functionality annotations of the genes/gene products with more specific contexts, such as the types of cellular locations or cells in which these biological functionalities were observed [31]. Similar calls for representing complex structures in linguistic annotation are also being made, for example representing semantic frames composed from other annotations [34]. In our own work, the next layer of annotation planned for the CRAFT corpus will also require the ability to dynamically construct concepts for use in assertional annotation [8]. To capture such information, annotations must point to knowledge structures more complex than singular concepts. We have represented two fundamental types of annotations: resource annotations, each of which points to a single RDF resource, and graph annotations, each of which points to an RDF graph encapsulating one or more RDF statements. Using our model, a user can create any combination of resource annotations and/or graph annotations, as motivated by the complexity of information that is sought to be captured in a given annotation effort.

#### Documenting the composition of annotations

As the annotations become more complex annotators (both human and computational) need the ability to refer to existing annotations as the basis of more complex annotations. While ontologies and other vocabularies used for annotation strive to be complete, it is likely that specific applications will require dynamic construction of concepts, either through data-driven methods [7,14]. or compositional concept formation [8].

Natural language processing (NLP) pipelines are frequently composed of sequences of components that each produce output based on the output of earlier annotation layers. For example, the output of a tokenizer might be fed into a part-of-speech detector, and both of which are fed into a named-entity recognizer, each component of which is dependent on the output of some or all of the previous components. There are generalized frameworks for building annotation pipelines, such as UIMA [35,36]; however these pipelines do not provide standardized models for documenting the compositional provenance of annotations. Other systems for language understanding, such as Direct Memory Access Parsing systems [37,38] including OpenDMAP [39] and REDMAP [24], use hierarchical patterns to compose semantic annotations. These systems produce knowledge structures that are analogous to those presented in Figure 2; however, they have no standard methods for documenting provenance. All of these use cases are covered in a generalized way by our model, which enables the tracking of both coarse-grained, annotation-level provenance and fine-grained, statement-element-level provenance.

Understanding the genetic basis of disease is a major focus of current biological and bioinformatics research and requires the integration of numerous types of annotation. For example, *epistasis* captures interactions between genes that affect function and phenotype, and *compositional epistasis* has been introduced [40] as a way to model multiple genes affecting a phenotype. A typical method for identifying epistasis starts with annotations of SNPs (single-nucleotide polymorphisms) and then applies a procedure for determining interactions among them [41,42]; the inputs to such procedures are SNP annotations and the outputs can be modeled as a higher-order annotation over the genome sequence that connects two or more of the SNP annotations. Capturing the dependency of the epistasis relation on the prior SNP annotation is important; SNP identification (variant calling) is a process that is dependent on the initial sequencing and assembly, the reference genome, and other factors and as such the SNP annotations of a genome may vary with different analyses. Our model would enable identifying and distinguishing epistasis relationships determined on the basis of one variant analysis from those based on another analysis performed under differing conditions.

#### Analyzing annotation errors and attributing blame

A common use of provenance information is for error analysis and blame attribution tasks. For example, if an annotation is deemed incorrect, the method by which that annotation was constructed needs to be investigated. This often starts with identifying all the annotations that contributed to its generation. It is possible that a lower-level annotation is incorrect and its use alone led to the larger annotation being incorrect. In the case of DMAP-style pattern recognition, this type of analysis is critical both for debugging during development as well as for analysis of results such as that done in the evaluation of REDMAP [24]. For example, in that evaluation it was important to identify whether errors were due to named-entity recognizers improperly identifying entities in the text or to larger patterns being improperly applied. Working in the opposite direction, if a lower-level annotation is deemed incorrect, it is important to identify all downstream annotations that are based on that annotation so that they too can be identified as incorrect and retracted. (Please see later section titled “Querying Using the Model” for examples of SPARQL queries that extract this type of provenance information using our model.)

### Efficiency of the model

Modeling statement-element-level provenance comes with the cost of reifying the statements and statement elements. In the worst case, this cost is 10 triples per statement in the graph annotation, or 14 triples if inferable type triples are explicitly represented as well: 7 triples are required to reify the statement, and 1 triple is needed each for the subject, property, and object of the statement to record its provenance. (However, in our experience, it is rare to record provenance for the property.) For example, as rendered in Figure 4, ga1 requires 2 triples for the annotation (3 with type information), 4 triples to record annotation-level provenance, and 9 triples (13 with types) to record the statement-element-level provenance. In contrast in the OA model [3] it takes 7 triples per text span to anchor an annotation to a piece of text. If the text has multiple spans there is an additional 2-triple overhead. To connect ga1 to text in the OA model requires 16 triples, 7 triples for each of the two text spans plus 2 triples of overhead for having multiple spans. Table 1 shows the number of triples required to model the example graph annotations used in this document and their provenance. It also shows the number of triples required to anchor these annotations to their corresponding spans of text using the OA model. In terms of counts of RDF triples required, it can be seen that recording statement-element-level provenance is comparable to associating annotations with text.

**Table 1:** Counts of triples required to represent annotations

**Annotation**	**Annotation triples**	**Annotation provenance triples**	**Statement element provenance triples**	**Text span triples**
ga1	2 (3)	4	9 (13)	16
ga2	3 (4)	2	16 (24)	16
ga3	6 (7)	3	32 (48)	30

Counts of triples required to represent annotations (and total counts including inferable type triples), their statement-element-level provenance, and their associated text spans.

We aimed in this work to lay down a low-level foundation for annotation provenance, which can then serve as the building blocks for higher-level models or axiomatization. We acknowledge that the use of reification to explicitly identify all of the low-level parts in our model leads to the production of additional triples. However, as more support for reasoning with axiomatizations in triple stores becomes available, extensions and abstractions of our model that reduce the counts of triples required could be defined. For example, if triples are reused from one graph to another, as is the case for statements s2 and s3 in Figure 4, a relation such as copyOf could be defined and used to directly connect these statements so as to obviate the need to reify and relate all of the corresponding RdfStatementElement instances from both statement triples. As the model is applied in practice, other patterns may emerge that point to additional optimizations or refinements. Mappings could also be constructed from our model to nanopublications [43,44] or RDF Molecules [45] to potentially reduce the number of redundant triples.

### Querying using the model

Due to the manner in which the model was integrated with the Relation Ontology (RO) and the Information Artifact Ontology (IAO), querying of the model can be quite straightforward in SPARQL. A common provenance-tracking task might be to identify all the annotations that are based on a given annotation that is suspected of being incorrect. For example, the following SPARQL 1.1 query returns all of the annotations that are based on ra7:

Similarly, a researcher may be interested in all of the *statements* that are based on a given annotation. If these RdfGraphAnnotation instances have had their statement-element-level provenance recorded using our model, such statements could be queried for directly. For example, the following SPARQL 1.1 query would retrieve all statements each of which has at least one statement element that is based on resource annotation ra7:

The first three namespaces are needed as part of the annotation model, and the last three are specific to the example domain and only used for more conveniently rendering the results. While this query and the one before are modeled using SPARQL 1.1 property paths, there is nothing in our model that requires them for querying. For example, using SPARQL 1.0, the property paths could be expanded using blank nodes or variables that are not captured in the results.

### Related work

Efforts in the representation of more structured annotations have tended to be idiosyncratic, specific to a particular type of annotation or task, and not broadly interoperable. For example, for the task of Gene Ontology (GO) annotation, in which the functionalities of genes and gene products represented in biomedical databases are associated to GO terms [46], the Gene Association File format (GAF 2.0) [31] enables the representation of constraints on the context in which a given annotation might be valid (*e.g.*, the type of cell in which the functionality is asserted to be present); however, this format is specific to this narrow task. Analogously, the corpus and computational linguistics communities have developed solutions for representing complex syntax and semantics for documents, *e.g.*, the Penn Treebank format [15], but these representations are mostly idiosyncratic and not interoperable. The Linguistic Annotation Framework (LAF) [34], along with its XML-based serialization GrAF [47] and the RDF-based representation DADA [48], allow for the markup of a wider range of linguistic phenomena, but they only permit the specification of functional (single-valued) properties. POWLA [49] is another linguistic corpora annotation formalism based in RDF and OWL, but, like LAF/GrAF, it introduces an ambiguity by using the same identifier to anchor information about both the annotation and what it denotes; hence, the formalism cannot clearly capture which information applies to the annotation and which applies to the denoted knowledge [50].

Several prominent efforts have focused on general representations of annotation, notably the Open Annotation (OA) model [3] and the Annotation Ontology (AO) [4]. These models represent three basic pieces of information for a given annotation: the thing being annotated (*e.g.*, a span of text), the denoted knowledge representation (*i.e.,* the concept or set of assertions denoted by the annotation), and the annotation itself, which connects the other two. These three things can be broadly aligned across the two models as well as with our model for the linkage of annotations to their denoted knowledge representations. A proposed integration of our model with the Open Annotation model can be found in Additional file 1 and an integration with the Annotation Ontology model in Additional file 2. In this paper, we have elided discussion of metadata such as author and creation date as well the connection of annotations to their respective targets, and our annotation model makes no constraints or requirements as to how these pieces of information are represented. The relations used by the Open Annotation model and the Annotation Ontology would both work well, and for most of such metadata the two models largely capture the same details. Though constructs that can denote complex knowledge structures have very recently been added to these models, there have been no mechanisms put forth by which complex annotations can be composed of more atomic annotations with their provenance unambiguously recorded.

Both the OA and AO annotation models seem to support one annotation pointing to multiple targets. However, it is ambiguous as to whether the annotation applies equally and independently to each target (*e.g.,* as for an annotation targeting the text spans of multiple mentions of “STAT6” in a piece of text with the corresponding Protein Ontology class (PR:000001933)) or if it is the union of the targets that is being annotated (*e.g.,* as for an annotation targeting the two discontinuous spans of text “c-terminal” and “tails” from the phrase “c-terminal cytoplasmic tails” with the Sequence Ontology class c_terminal_region (SO:0100015) [51]). We strongly assert that an annotation with multiple targets should be interpreted as a single discontinuous annotation and that the alternate shared-annotation interpretation should be disallowed by all models. On its surface, the shared-annotation interpretation seems to be beneficial in that it saves triples and seems easier to create. However, it muddles information represented for the purposes of provenance tracking or error analysis; for example, if three out of four of the shared targets for an annotation are correct, but the fourth target is incorrect, this information could not be accurately captured. Furthermore, in the case of compositional annotations, it could not be clearly represented which of the shared annotations and targets are connected via basedOn links and which are not. When considering increasingly complex annotations and how annotations will be used by downstream applications and models, it is clear that one annotation to one Annotation instance is the only lossless approach.

Numerous other models of triple-level provenance also exist, for example, PaCE [52] and RDF coloring [53], but these models require more complicated URI-minting procedures and systems that can understand the compositional URIs they produce. The most related triple-provenance model is that for nanopublications [43,44], which is compatible with our GraphAnnotation model in that it provides a method for recording triple-level provenance and annotating sets of triples with metadata. However, the primary purpose of nanopublications is to enable attribution and validation of scientific statements, and as such it does not model resource annotations, targeting annotations to other content such as text, or fine-grained statement-element-level provenance. Our approach is complementary to microattribution proposals to attribute data such as disease-implicated genetic variants to the scientists who determine them [54].

Also related to our research is work being done by the scientific workflow provenance community. Proof Markup Language [55] models the justifications of reasoning results from Semantic Web services, while work such as Provair [5] aims to document work-flow provenance. Trust and authenticity are also active areas of research [56]. Through provenance workshops [57] and challenge meetings [58], the Open Provenance Model (OPM) [59] has been developed. Other community efforts have led to the creation of the PROV [60] model, which provides a data model for building representations of the entities, people and processes involved in producing a piece of data or thing. A proposed integration of our provenance relations with the object-centric portion of PROV-O (the OWL-ontology version of PROV) [61] is provided in Additional file 4.

## Conclusions

We have presented a model for representing compositional annotations and annotation provenance, and provided examples of application areas for the model. The model can be used to link annotations to their denoted knowledge representations, and we have divided the annotation space into resource annotations, in which RDF resources are used to annotate targets, and graph annotations, in which graphs composed of one or more RDF triples are used. With this model, progressively more complex annotations can be composed from other annotations, and this provenance can be unambiguously represented at either a coarse- or fine-grained level. We have designed our annotation model to be generic so as to facilitate the concurrent use of multiple types of annotations. Additionally, it allows for the creation of arbitrarily complex annotations, both in terms of their denoted knowledge and of any other annotations upon which they rely. All of this information can be losslessly recorded, thus facilitating inference and error tracking in large computational annotation efforts. We have provided an OWL representation of our model integrated with the Information Artifact Ontology, as well as proposed integrations with the Open Annotation model, the Annotation Ontology, and the PROV Ontology.

## Endnotes

^a^Although activation (mentioned in the example sentence fragment in Figure 2) is semantically narrower than positive regulation, we use the GO class positive regulation of biological process here for simplicity, as there is no more specific subclass in the GO that generically represents the activation of a biological process. Similarly, inhibition is semantically narrower than the GO class negative regulation of biological process.

^b^ The OBO Relation Ontology, upon which the ontologies of the OBO library rely, uses the obo: namespace. We extend the Relation Ontology using the namespace kro:.

^c^ While relations are typically named as verbs or verb phrases, we modeled these relation names to be analogous to the core RDF statement model.

## Competing interests

The authors declare that they have no competing interests.

## Authors’ contributions

KV and KML conceived the project. KML and MB were the primary developers of the proposed models. KV contributed to the text-mining use cases and to the alignment with existing annotation models. LEH contributed to the alignment with IAO and other OBO efforts. KML, MB, and KV contributed to the writing of the manuscript. All authors reviewed and approved the work.

## Supplementary Material

Additional file 1 **Appendix A.** Alignment with the Open Annotation Model. Appendix describing an alignment from the proposed model to the Open Annotation model. Click here for file

Additional file 2 **Appendix B.** Alignment with the Annotation Ontology. Appendix describing an alignment from the proposed model to the Annotation Ontology model. Click here for file

Additional file 3 OWL file containing proposed model as an extension of the IAO. Click here for file

Additional file 4 **Appendix C.** Alignment with the PROV Ontology. Appendix describing an alignment from the proposed model to the PROV OWL model. Click here for file
