# Supplementary material for: Representing annotation compositionality and provenance for the Semantic Web
Source: J Biomed Semantics. 2013 Nov 22;4:38. doi: 10.1186/2041-1480-4-38 (PMC4129183; doi:10.1186/2041-1480-4-38)
Supplement: Additional file 1 — Appendix A. Alignment with the Open Annotation Model. Appendix describing an alignment from the proposed model to the Open Annotation model. [file 2041-1480-4-38-S1.pdf]

Appendix A: Alignment with the Open Annotation Model

The Open Annotation model (http://www.openannotation.org/spec/core/) and its extension (http://www.openannotation.org/spec/extension/) represent three basic pieces of information for an annotation: the annotation target (*i.e.*, what is being described), the annotation body (*i.e.*, the description of the target), and the annotation itself. As our model focuses on annotations, their denoted knowledge representations, and the provenance of these, we present an alignment with the annotation and body portions of the OA model. We place no requirements on the target representation (although see discussion in Related Work section). The following is an RDFS alignment of our model with the OA model in N3 notation.

```
@prefix kiao: <http://kabob.ucdenver.edu/iao/>
@prefix oa:   <http://www.w3.org/ns/openannotation/core/>
@prefix rdfs: <http://www.w3.org/1999/02/22-rdf-syntax-ns#>
@prefix rdfg: <http://www.w3.org/2004/03/trix/rdfg-1/>
```

The class `oa:Annotation` is more specific than `kiao:Annotation` and more general than `kiao:RdfResourceAnnotation` and `kiao:RdfGraphAnnotation`, thus the following relations hold:

```
oa:Annotation               rdfs:subClassOf kiao:Annotation.

kiao:RdfResourceAnnotation  rdfs:subClassOf oa:Annotation.
kiao:RdfGraphAnnotation     rdfs:subClassOf oa:Annotation.
```

If OA annotations are being converted to KIAO annotations, there are several ambiguities. Primarily the OA definitions place no cardinality constraints on `oa:hasBody` or `oax:hasSemanticTag` (relations used to map an annotation to its denoted knowledge representation); one annotation can contain multiple of assertions using each relation. In such cases, each assertion in the OA model should likely be converted into an independent annotation in the KIAO model. The

`oax:hasSemanticTag` property is a more specific type of `iao:denotes`, and annotations using this property should be converted into instances of `kiao:RdfResourceAnnotations`.

```
oa:hasSemanticTag rdfs:subPropertyOf iao:denotes.
```

It is unknown if the object of `oa:hasBody` is an `rdfs:Resource` (which should translate an annotation instance of the class `kiao:RdfResourceAnnotation`) or an `rdfg:Graph` (which should translate to an annotation instance of the class `kiao:RdfGraphAnnotation`). We can assert that `oa:hasBody` is also a subproperty of `iao:denotes`:

```
oa:hasBody rdfs:subPropertyOf iao:denotes.
```

Thus, each `oa:hasBody` assertion translates to an `iao:denotes` assertion.

For the conversion of KIAO annotations to OA annotations, one may think that `iao:denotes` could straightforwardly be made subproperty of `oa:hasBody`:

```
iao:denotes rdfs:subPropertyOf oa:hasBody.
```

Thus, each `iao:denotes` assertion would translate to an `oa:hasBody` assertion. However, `iao:denotes` is defined to hold not only among annotations but more generally among information content entities, while `oa:hasBody` only pertains to annotations. If this translation was broadly accepted, it is possible that `iao:denotes` assertions pertaining to information content entities other than annotations would be erroneously converted to `oa:hasBody` assertions, which would necessarily pertain to annotations. The only generally correct translation is to convert all `iao:denotes` assertions *for annotations only* (*i.e.*, with annotations as the subjects of the assertions) to `oa:hasBody` assertions. Using the OA extension model, it would also be acceptable to convert the `iao:denotes` assertions from instances of

`kiao:RdfResrouceAnnotation` to assertions using the property

`oax:hasSemanticTag`.
