# Supplementary material for: Representing annotation compositionality and provenance for the Semantic Web
Source: J Biomed Semantics. 2013 Nov 22;4:38. doi: 10.1186/2041-1480-4-38 (PMC4129183; doi:10.1186/2041-1480-4-38)
Supplement: Additional file 2 — Appendix B. Alignment with the Annotation Ontology. Appendix describing an alignment from the proposed model to the Annotation Ontology model. [file 2041-1480-4-38-S2.pdf]

Appendix B: Alignment with the Annotation Ontology

The Annotation Ontology (http://code.google.com/p/annotation-ontology/) represents three pieces of information for an annotation: the annotation context (*i.e.*, what is being described), the topic (*i.e.*, the description of the context), and the annotation itself, which connects the other two. As our model focuses on annotations, their denoted knowledge representations, and the provenance of these, we present an alignment of our model with the annotation and topic portions of the AO. We place no requirements on the representation of context (although see discussion in Related Work section). The following is an RDFS alignment of our model with the AO in N3 notation.

```
@prefix kiao: <http://kabob.ucdenver.edu/iao/>
@prefix ao:   <http://purl.org/ao/core/>
@prefix aot:  <http://purl.org/ao/types/>
@prefix rdfs: <http://www.w3.org/1999/02/22-rdf-syntax-ns#>
@prefix rdfg: <http://www.w3.org/2004/03/trix/rdfg-1/>
```

The class `ao:Annotation` is a more specific kind of `kiao:Annotation` while `kiao:RdfResourceAnnotation` and `kiao:RdfGraphAnnoation` are more specific than `ao:Annoation`, thus the following relations hold:

```
ao:Annotation               rdfs:subClassOf kiao:Annotation.

kiao:RdfResourceAnnotation  rdfs:subClassOf ao:Annotation.
kiao:RdfGraphAnnotation     rdfs:subClassOf ao:Annotation.
```

AO also has several more specific annotation types that could be aligned individually with our model; however, the semantic implications of doing so are not clear. In particular, the AO has the class `aot:Qualifier`, which is used to “express a relationship between the object of the annotation and a well-defined semantic entity.” In our model, `kiao:RdfResourceAnnotation` meets that definition, so we can at least state that `kiao:ResourceAnnotation` is a subclass of `aot:Qualifier`:

```
kiao:ResourceAnnotation rdfs:subClassOf aot:Qualifier.
```

It is unclear if the inverse relation could be made as well, in which case `kiao:ResourceAnnotation` and `aot:Qualifier` would be equivalent.

`kiao:RdfGraphAnnotation` and `ao:GraphAnnotation` can be declared to be equivalent:

```
kiao:RdfGraphAnnotation rdfs:subClassOf ao:GraphAnnotation.
ao:GraphAnnotation rdfs:subClassOf kiao:RdfGraphAnnotation.
```

If AO annotations are being converted to KIAO annotations, there are several ambiguities. AO has two relations `ao:hasTopic` and `ao:body`. When an AO annotation has both relations it is unclear what are the intended semantics, and it is likely each would convert independently into KIAO annotaitons. The relation `ao:hasTopic` implies that annotation should be translated into a `kiao:RdfResrouceAnnotation` with a corresponding `iao:denotes` assertion. We can assert that `ao:hasTopic` is also a subproperty of `iao:denotes`:

```
ao:hasTopic rdfs:subPropertyOf iao:denotes.
```

Thus, each `ao:hasTopic` assertion translates to an `iao:denotes` assertion.

The relation `ao:body` is underspecified. This relation can have as an object a `Resource`, a `Graph`, or even literal strings. In the context of an `ao:GraphAnnotation` it is assumed this relation points to a `Graph`, and thus in this context only `ao:body` could be translated to a `kiao:RdfGraphAnnotation` with a corresponding `iao:denotes` assertion to the graph. In all other contexts the meaning of this relation cannot be unambiguously inferred, although it can generally translated into an `iao:denotes` assertions (expect in the case of string literals).

For the conversion of KIAO annotations to OA annotations, one may think that `iao:denotes` could straightforwardly be made subproperty of `ao:body`:

```
iao:denotes rdfs:subPropertyOf ao:body.
```

Thus, each `iao:denotes` assertion would translate into a `ao:body` assertion. However, `iao:denotes` is defined to hold not only among annotations but more generally among information content entities, while `ao:hasTopic` and `ao:body` only pertains to annotations. If this translation was broadly accepted, it is possible that `iao:denotes` assertions pertaining to information content entities other than annotations would be erroneously converted to `ao:hasTopic` or `ao:body` assertions, which would necessarily pertain to annotations. The only generally correct translation is to convert all `iao:denotes` assertions *for annotations only* (*i.e.*, with annotations as the subjects of the assertions) to `ao:body` assertions. In the context of instances of `kiao:RdfResourceAnnotation` it is likely more appropriate to convert the corresponding `iao:denotes` assertion into a `ao:hasTopic` assertion.
