# Supplementary material for: Representing annotation compositionality and provenance for the Semantic Web
Source: J Biomed Semantics. 2013 Nov 22;4:38. doi: 10.1186/2041-1480-4-38 (PMC4129183; doi:10.1186/2041-1480-4-38)
Supplement: Additional file 4 — Appendix C. Alignment with the PROV Ontology. Appendix describing an alignment from the proposed model to the PROV OWL model. [file 2041-1480-4-38-S4.pdf]

Appendix C: Alignment with the PROV Ontology

The PROV Ontology model in OWL (PROV-O, http://www.w3.org/TR/prov-o/) is a general model of provenance that represents event-oriented as well as data-centric provenance. As our model is data-centric, we present an alignment of our model with the data-centric portion of the more general PROV-O model. The following is an RDFS alignment of our model with the PROV-O in N3 notation.

```
@prefix kiao: <http://kabob.ucdenver.edu/iao/>
@prefix prov: <http://www.w3.org/ns/prov#>
@prefix rdfs: <http://www.w3.org/1999/02/22-rdf-syntax-ns#>
```

Both `kiao:Annotation` and `kiao:RdfStatementElement` (and even the IAO information content entity class) can be declared subclasses of `prov:Entity`:

```
kiao:Annotation       rdfs:subClassOf prov:Entity.
kiao:RdfStatementElement rdfs:subClassOf prov:Entity.
```

`kiao:basedOn` assertions both at the statement level and the statement-element level can be represented as `prov:wasDerivedFrom` assertions. `kiao:basedOn` can be declared a subproperty of `prov:wasDerivedFrom`. since our model is specific to information content entities:

```
kiao:basedOn rdfs:subPropertyOf prov:wasDerivedFrom.
```

PROV-O also defines `prov:wasQuotedFrom` as a subproperty of `prov:wasDerivedFrom`, which is to be used when an entity is derived by being copied directly. This property more directly models the type of provenance that is being recorded in our examples of statement-element-level provenance. Its use could help distinguish when statement elements are copied as opposed to derived in some other way. One may think that it would be beneficial to assert the following:

```
prov:wasQuotedFrom rdfs:subPropertyOf kiao:basedOn.
```

However, `kiao:basedOn` is information content entity specific, while the PROV relation is not. This would cause `prov:wasQuotedFrom` to be unnecessarily and incorrectly restricted to only information content entities instead of any `prov:Entity`. If converting from PROV assertions to KIAO assertions, the only generally correct translation is to assert each `prov:wasQuotedFrom` assertion between two `kiao:RdfStatementElement` instances as a `kiao:basedOn` assertion.
